# The *General* Age of Leadership: Older-Looking Presidential Candidates Win Elections during War

**DOI:** 10.1371/journal.pone.0036945

**Published:** 2012-05-23

**Authors:** Brian R. Spisak

**Affiliations:** Department of Social and Organizational Psychology, VU University, Amsterdam,The Netherlands; University of Utah, United States of America

## Abstract

As nation-state leaders age they increasingly engage in inter-state militarized disputes yet in industrialized societies a steady decrease in testosterone associated with aging is observed – which suggests a decrease in dominance behavior. The current paper points out that from modern societies to Old World monkeys increasing both in age and social status encourages dominant strategies to maintain acquired rank. Moreover, it is argued this consistency has shaped an implicit prototype causing followers to associate older age with dominance leadership. It is shown that (i) faces of older leaders are preferred during intergroup conflict and (ii) morphing U.S. Presidential candidates to appear older or younger has an overriding effect on actual election outcomes. This indicates that democratic voting can be systematically adjusted by activating innate biases. These findings appear to create a new line of research regarding the biology of leadership and contextual cues of age.

## Introduction

Recent political science data has found that leaders in industrialized society are in general more likely to initiate and be the target of militarized disputes as their age increases [1]–[3]. This is curious considering the apparent contradiction with decreasing testosterone levels associated with aging in Western society [4] which would imply a reduction of competitive behavior among older individuals [5]. Do older leaders somehow have a special role to play during intergroup conflict and are followers implicitly aware of this connection? Converging literature from multiple disciplines highlights a positive connection between increasing age of a leader, their status, and the use of dominant group strategies to maintain the acquired benefits of their position. It is argued that followers are implicitly aware of this connection and will endorse this older *dominant* leadership to address intergroup conflict.

Humans are among a number of species that have adopted a social group strategy [6], and consequently face problems of coordination. In turn this creates a selection pressure (across species) for efficient group behavior [7] such as an ability to lead and follow [8] and form cognitive leadership prototypes comprised of traits best suited to assume specific leadership roles [9]. These cognitive leadership prototypes are a human social function occurring automatically as a method for expediting leadership emergence for specific coordination problems [10]. Intergroup conflict (war) is one such reoccurring problem with the ability to shape human behavior [11].

How then has age potentially helped to mold this dominant leader prototype? Cross-species evidence supports an evolutionarily consistent pattern of older age, increasing status, and dominance behavior around which an adaptive leader prototype can form. A study of male Japanese Macaques found that those categorized as “old-male” (15–20 yr old) tended to occupy higher dominance ranks until they reach the “ancient” age group [12]. Similarly, male Chimpanzees tend to rise in rank as they age until senescence inhibits the maintenance of status via physical dominance [13]–[15]. Chimpanzees higher in rank also tend to act more aggressively [16]. From an anthropological perspective, it appears a version of this strategy resonates in human behavior. In prestate societies age is positively associated with dominant coalition structures to maintain status [17].

This combined evidence corresponds nicely with the observed dominance patterns of older industrialized leaders [1]–[3] and suggests this age-based status via dominance connection is a persistent configuration. Given this consistency, it is likely that older age has become part of the intergroup conflict prototype. Assuming that group members benefit most from correctly assigning leadership [8], then followers under threat should interpret older cues as signals of dominance and thus as an adaptive leadership style to solve intergroup conflict situations.

One such age cue that may have helped form this war-leader prototype is the human face. Like sex, age is a basic classification [18]. Children as young as 4 months can discriminate facial signals of age, and neurological research has found evidence for age recognition of the human face even when other capabilities such as facial identity and emotion recognition are lost as a result of brain injury [19]. Thus, age classification is a fundamental method for interpreting social information and consequently contributes to the development of cognitive representations such as the leader prototype for conflict.

Given the evolutionarily consistent threats and opportunities associated with intergroup conflict, the apparent selection pressure for older high status individuals to maximize their success via dominant strategies, the tendency to form context-specific leadership prototypes to increase group effectiveness, and the robust neurological propensity to make facial age distinctions, it is argued that signals of older age have been selected for as part of an adaptive intergroup conflict leadership prototype.

It is hypothesized that followers will make a distinction between relatively older and younger leader candidates and consistently endorse older-looking leaders during intergroup conflict as an outcome of the match between perceived dominance leadership and requirements of war. In addition, it is predicted that these old/young leader distinctions have real consequences. By morphing the facial features of Barack Obama and John McCain to look respectively older and younger, it is expected that during a time of intergroup conflict followers will vote in-line with general cues of relative older age regardless of the underlying personal features. Finally, to ensure that this phenomenon is about dominance and not merely a matter of general experience it is anticipated that the ability for older leaders to gain acceptance will be significantly reduced when the situation requires peaceful cooperation. For all experiments written consent was obtained from all participants and the research was approved by the ethics committee in the School of Psychology at the University of Kent.

## Experiment 1

### Methods

#### Participants

For this online experiment 145 participants (97 females and 48 males; *M*_age_ = 22.83, *SD* = 6.02) volunteered.

#### Materials and procedure

**Figure 1:**
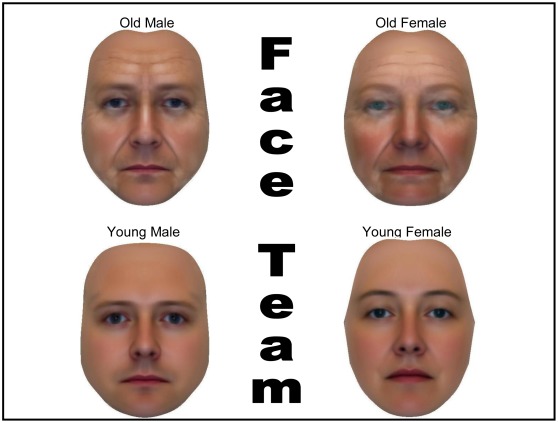
Old and young face teams: example of stimuli.

Using facial composite software [20] both male and female composite faces were created in older and younger trajectories. This provided a face team comprised of one younger male, one older male, one younger female, and one older female (Fig. 1). Five such teams were created to minimize the idiosyncratic effect of any one grouping thereby increasing the reliability of isolating the age cue.

Participants were presented with pairs of faces side-by-side. The four face-types (age x gender) were paired in all possible combinations creating six head-to-head voting decisions per scenario. Participants were presented with an intergroup nation-level war scenario and asked to vote for one of the two faces they preferred as president. The procedure continued until participants voted for all six head-to-head face pairings in the scenario. This repeated measure design imbeds the variable of interest (i.e., age) in combination with a distracter variable (i.e., gender). To further distract participants three filler scenarios were incorporated. Finally, for control purposes, it was examined if an intergroup peace scenario would produce a reverse in voting patterns to war - though the theoretical argument does not make any specific predictions for peace leadership other than that dominant signals associated with older leaders would not be preferred (see Appendix S1 for experimental scenarios). In total, participants were presented with two experimental scenarios (war and peace), three filler scenarios, and had to make 6 head-to-head voting decisions per scenario.

Participants were provided with a link to a secure website and logged on to start the experiment. They were shown an informed consent page, asked demographic questions, and given instructions. Following the introduction, participants clicked the start button and a pop-up window appeared with the scenario. Once the scenario was read they clicked an “OK” button to proceed. This pop-up window occurred between each scenario to ensure the participants were aware of the changing situation. Upon clicking “OK” participants were presented with one of the paired face combinations side-by-side with a “vote” button below each face. The scenario also remained on the screen. Participants voted for each of the face pairings (i.e., six pairings) for each scenario. Order of the scenarios, face teams, face pairings, and side of the screen the faces appeared were randomized to eliminate order effects. Upon completion participants were debriefed and thanked for participation.

### Results and Discussion

To investigate voting preferences for older versus younger facial features analysis was conducted within and between war and peace scenarios using a Bradley-Terry Model. This particular version of the model accounts for the general preference for the judged objects (young-male face, old-male face, young-female face, and old-female face) as well as the interdependency of multiple paired comparisons within the same subject [21]. The model includes correlations between judgments in the analysis to account for overdispersion caused by clustering. The preferences are reparametrized to yield a 2 x 2 crossed design of age (the variable of interest), sex (the distracter variable), and the interaction between these factors.

As expected, it was found that older-looking candidates were significantly preferred during war (Wald χ^2^ (df = 1) = 21.15, *p*<.001). Analysis of the distracter variable (sex) yielded a main effect preference for male candidates (Wald χ^2^ (df = 1) = 17.44, *p*<.001) and *no* interaction (Wald χ^2^ (df = 1) = 0.55, *p* = .46). For the control scenario of intergroup peace younger candidates were preferred (Wald χ^2^ (df = 1) = 25.05, *p*<.001) which suggest there is *not* an overall bias towards older candidates. Also, the distracter variable had a main effect where female candidates were preferred for peace leadership (Wald χ^2^ (df = 1) = 10.73, *p* = .001) and *no* interaction between variables (Wald χ^2^ (df = 1) = 1.51, *p* = .22). Finally, gender of the participant was analyzed. For the war scenario there was a significant three-way interaction (Wald χ^2^ (df = 1) = 4.23, *p* = .04). This three-way interaction entailed that male participants did *not* discriminate between males and females if they were young, but preferred males above females when they were old. Female participants, on the other hand, did *not* discriminate between males and females if they were old, but preferred males above females when they were young. For the peace scenario there were *no* interactions with gender of participant (all *p*-values >.10).

In sum, novel evidence was found indicating that followers do make the association between older age and dominant leadership and this in turn influences their voting behavior. Further, if followers were simply associating older age with the need for experienced leadership in general the observed switch favoring younger leaders during peace would not be expected.

## Experiment 2

### Methods

**Figure 2:**
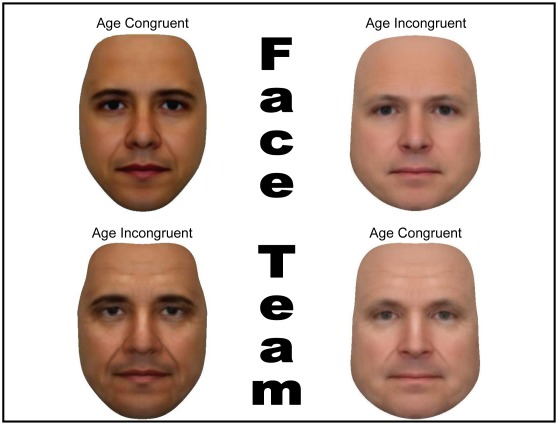
Age congruent and incongruent McCain- and Obama-like faces.

To test the hypothesis that these connections have actual relevance on modern elections facial features of Barack Obama and John McCain were morphed on unrecognizable young and old male faces (Fig. 2), thus creating a young and old Obama-like face and a young and old McCain-like face (conducted in August 2008 - prior to the U.S. Presidential elections).

#### Participants

This online experiment was conducted by 224 participants (111 male, 100 female, and 13 decline; *M*_age_ = 24.14, *SD* = 10.71, and 20 decline) who volunteered and were provided with informed consent.

#### Materials and procedure

Three neutral expression images were gathered on the Internet of both McCain and Obama. The three images each were averaged together to create an average face for both candidates – thus reducing the idiosyncratic effect of any one photograph. Then 45% of both images were morphed with neutral younger and older composite facial templates. This procedure yielded four separate images; one *young* Obama-like face, one *old* Obama-like face, one *young* McCain-like face, and one *old* McCain-like face (Fig. 2).

The exact testing procedures for Experiment 1 were used with the exception that all facial morphs were male images given that both candidates were male. As before, participants were debriefed and thanked for their participation.

### Results and Discussion

The results indicated that older age has a strong effect on voting success and leadership emergence during war (Wald χ^2^ (df = 1) = 45.97, *p*<.001) and though a weak but significant effect was found in favor of Obama-like faces (Wald χ^2^ (df = 1) = 3.93, *p* = .05) when the individual voting patterns for all six head-to-head competitions were considered using chi-squares the voting exactly followed an older  =  conflict leader pattern (Table 1). Specifically, regardless of being an Obama-like face or McCain-like face, participants selected the candidate with the older face. In addition, when the Obama- and McCain-like faces shared the same age category (i.e., old-old or young-young) voting percentages did not significantly differ. If an overwhelming preference for an Obama- or McCain-like face did exist this outcome would not occur. In the control scenario of intergroup peace it appeared that the Obama-like was preferred (Wald χ^2^ (df = 1) = 14.38, *p*<.001) though, as in Experiment 1, a main effect endorsing younger leaders was detected (Wald χ^2^ (df = 1) = 5.94, *p*<.05). Finally, for both scenarios, there were *no* interactions with gender of participant (all *p*-values >.10).

**Table 1:** Forced-choice pairs voting results for the Obama- and McCain-like facial images during intergroup conflict.

War Pairings(bold = older face image)	Older Face Winner*	Winning Percentage	*χ* ^2^	*p*
Obama young – **McCain old**	**✓**	35%v65%	20.64	.000
Obama young – McCain young	NA	51%v49%	0.16	.688
Obama young – **Obama old**	**✓**	30%v70%	36.16	.000
McCain young – **Obama old**	**✓**	33%v67%	24.45	.000
McCain young – **McCain old**	**✓**	30%v70%	36.16	.000
McCain old – Obama old	NA	44%v56%	3.02	.082

* Check marks indicate statistically significant wins.

The results from Experiment 2 provide evidence for the overall value and relevance of older age cues for leadership during intergroup war. For instance, even if participants made a connection between the morphed faces and actual candidates, the findings indicate that it did not influence the overall results of the mock elections – which one might expect if participants were merely voting for their actual preferred presidential candidate. Instead, they voted for the older candidate. This strongly suggests that McCain would have had (and future older candidates will have) an increased chance of securing leadership by making the threat of intergroup conflict the most salient situational concern in the minds of the voters – perhaps something McCain did *not* consistently do relative to other factors (for an overview of the 2008 U.S. Presidential elections see [22] and for an agent-based model incorporating actual polling data see [23]).

## Discussion

As hypothesized, it was found that indeed facial cues of relatively older age are associated with leadership emergence during times of intergroup conflict (not intergroup peacekeeping) and these age cues can have an impact on actual political candidates. These two experiments appear to be the first to provide such age- and situation-based evidence and clarify the apparent contradictions between political science data and hormonal research on testosterone. Namely, nation-state leader’s become more militarized with inter-state relations as they age [1]–[3], yet in industrialized society a steady decline in testosterone with increasing age is observed [4] which would indicate a reduction of dominant behavior [5].

It could be that throughout much of hominid evolution there was a positive correlation between age, status, and testosterone and this evolved cognitive leadership prototype for conflict is still being activated even when the hormonal response has shifted. Further, it may be that modern high status leaders do *not* experience this drop in testosterone with age. Also, at a proximate level, it may be that followers simply use age as a proxy for experience, and during turbulent times this is highly prized relative to leadership requirements during peace – which may allow for younger leaders to emerge in the absence of specific threats. However, the current results suggest not only are younger leaders afforded more opportunity during peace, they are systematically preferred. Future work will want to investigate these inferences by examining followership motivation as well as attaining hormone samples from CEO’s, high ranking military officials, and other such leaders and comparing their testosterone levels with population averages.

Existing data clearly indicates a status-based configuration associated with aging leaders and dominance is consistent (from Japanese Macaques to modern industrialized societies; [1]–[3], [12]). The experimental results further suggest followers are implicitly aware of this basic heuristic and when the situation is manipulated it alters voting behavior in the expected direction. Previous face perception work has shown the ability to predict election outcomes require minimal information [24], [25]. The current research extends past prediction by manipulating adaptive contingencies underlying followership behavior to systematically alter which candidate is victorious. Surprisingly, given this influence of age, there is a dearth of literature on the topic and how it relates to leaders [26]. Consequently these findings create a new avenue of research in the field of leadership with regard to age and context.

The results provide coherency across various fields and encourages further investigation into potential age-related differences in leadership perceptions, emergence, and behavior. Refinement of the current work will likely require incorporation of additional methodologies such as hormonal studies, neuroscience, and agent-based modeling. Also, this work has a wide range of applications from understanding how best to utilize younger and older leaders in organizations to identifying political candidates best suited for managing war or peace. These findings are crucial for understanding how the fundamentally democratic process of voting can be potentially disrupted by our innate biases.

## Supporting Information

Appendix S1 **Intergroup war and peace scenarios used for Experiments 1 and 2.**
(DOCX)
