# Supplementary material for: The General Age of Leadership: Older-Looking Presidential Candidates Win Elections during War
Source: PLoS One. 2012 May 23;7(5):e36945. doi: 10.1371/journal.pone.0036945 (PMC3359335; doi:10.1371/journal.pone.0036945)
Supplement: Appendix S1 — Intergroup war and peace scenarios used for Experiments 1 and 2. (DOCX) [file pone.0036945.s001.docx]

**Appendix S1**

War

Your country of Taminia is at war with the neighboring country of Robania. It has been an aggressive, costly, and competitive war with no side willing to concede. Recently, Robania has increased their forces and intensified their bombing raids. This has made everyone exceptionally concerned for their safety. You and your fellow citizens are determined to establish dominance over Robania in order to protect the lands, resources, and people of Taminia. Currently, your country is in the middle of a presidential election. Vote for a face in each pairing you think looks most like the leader in a war-time situation.

Peace - Between

Your country of Taminia has a longstanding and reciprocal relationship with the neighboring country of Rokland. This alliance, however, has become strained due to conflicting policies of your respective governments. Both sides are threatening to restrict trade and close the borders. Also, it has been rumored that military forces will be mobilized within the next six months. If that happens, war is almost certain. From the perspective of most Taminians, physical conflict is something that should be avoided at all cost. Instead, the people want to restore cooperation with Rokland through non-violent resolution that emphasizes creativity and understanding. Currently, your country is in the middle of a presidential election. Vote for a face in each pairing you think looks most like the leader to ensure peace and encourage connectivity between groups.
